# Supplementary material for: Tet1 and Tet2 maintain mesenchymal stem cell homeostasis via demethylation of the P2rX7 promoter
Source: Nat Commun. 2018 Jun 1;9:2143. doi: 10.1038/s41467-018-04464-6 (PMC5984622; doi:10.1038/s41467-018-04464-6)
Supplement: Supplementary file 1 — Supplementary Information [file 41467_2018_4464_MOESM1_ESM.pdf]

Supplementary Information

Tet1 and Tet2 maintain mesenchymal stem cell homeostasis via demethylation of the *P2rX7* promoter

Yang et al.

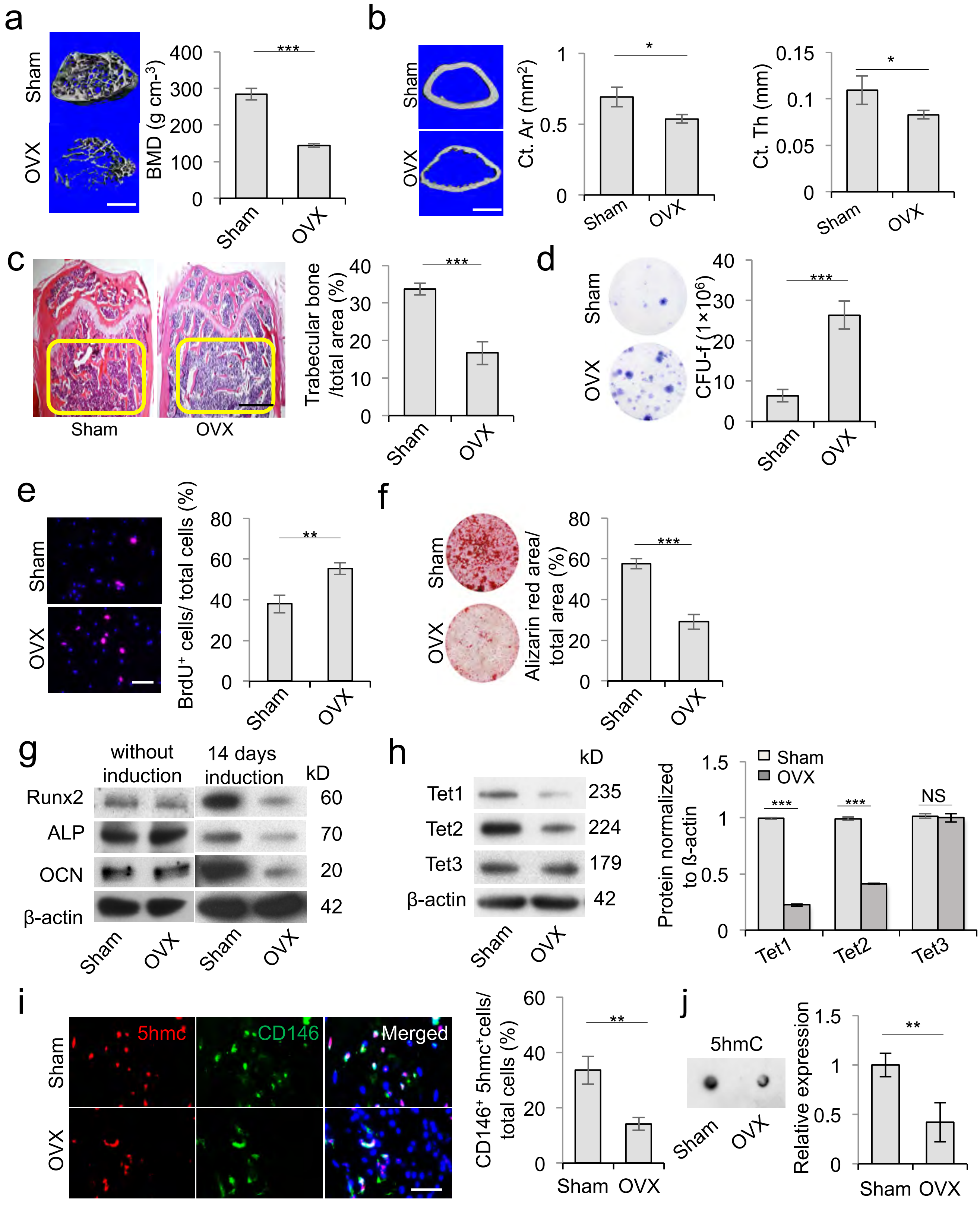

Supplementary Figure 1

Supplementary Figure 1. The expression levels of Tet1 and Tet2 are downregulated in OVX BMMSCs. (a) Micro-CT analysis showed bone mineral density (BMD) of trabecular bone area in the femurs of sham control and OVX mice. **(b)** Micro-CT analysis showed that the cortical bone volume in the sham control and OVX femur, as determined by cortical bone area (Ct.Ar) and cortical thickness (Ct.Th) of the femurs. **(c)** H&E staining showed trabecular bone volume (yellow-circled area) in the distal femurs of sham and OVX mice. **(d)** CFU-F of control and OVX BMMSCs were assessed by toluidine blue staining. **(e)** BrdU labeling assay showed proliferation rates of control and OVX BMMSCs. **(f)** When cultured under osteogenic inductive conditions, mineralized nodules formation of control and OVX BMMSCs, as assessed by alizarin red staining. **(g)** The expression of *Runx2*, *ALP* and *OCN* in control and OVX BMMSCs under 14-days osteogenic induction or not. **(h)** Western blotting showed the expression of Tet1, Tet2 and Tet3 in control and OVX BMMSCs. **(i)** 5hmC and CD146 double-positive cells was detected in control and OVX mouse bone marrow by immunostaining. **(j)** Global 5hmC expression in control and OVX BMMSCs, as assessed by dot blot assay. Age of 8-10 weeks C57BL/6J mice was used in these experiments. *P<0.05, **P< 0.01, ***P< 0.001. (mean ± SD). Scale bars, 400 μm (a, b), 1 mm (c), 50 μm (e), 25 μm (i). Results are from three independent experiments.

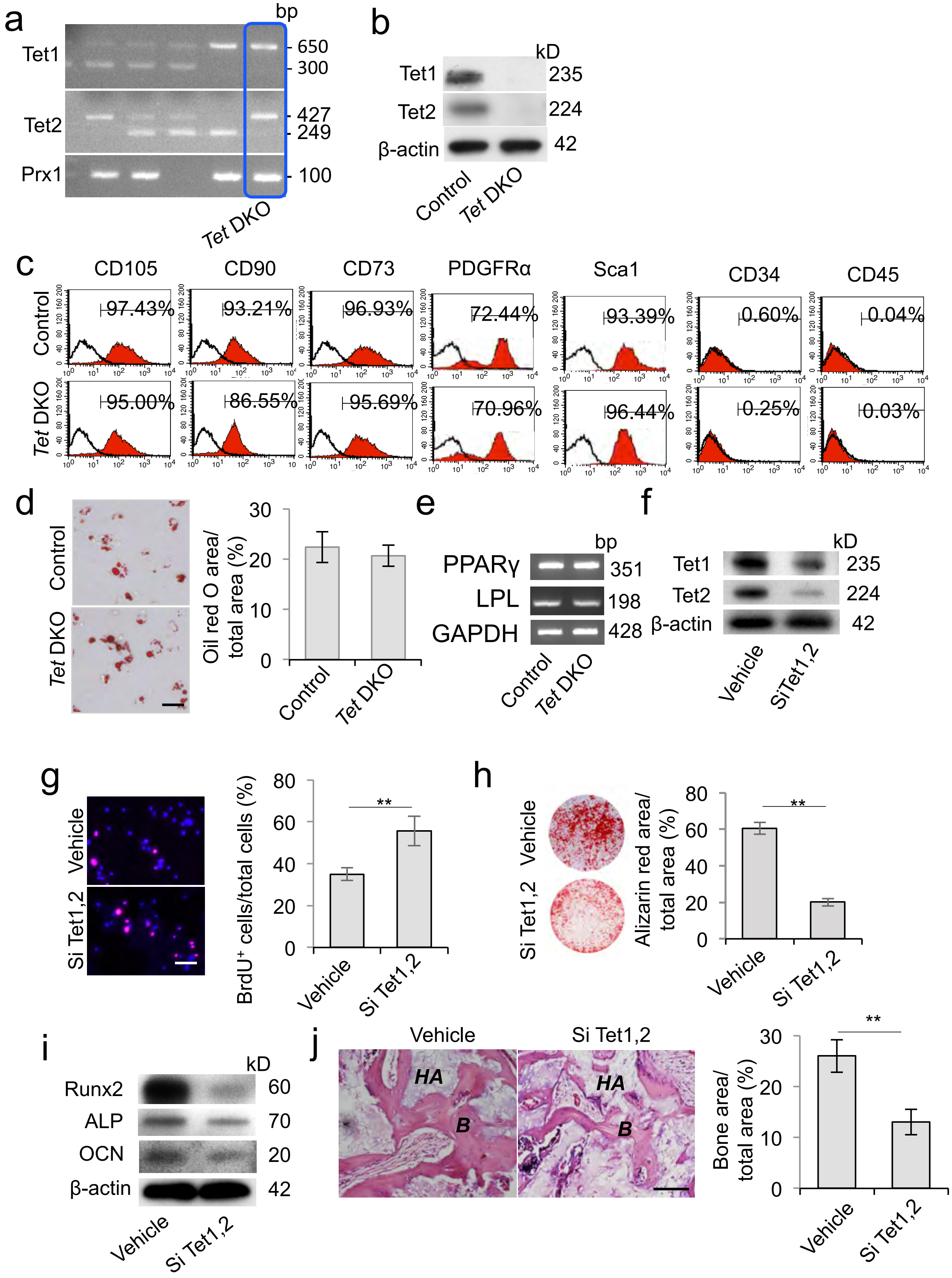

Supplementary Figure 2

Supplementary Figure 2. Tet1 and Tet2 knockdown impairs BMMSC function. **(a)** One representative image for genotyping of *Tet* DKO mice by PCR analysis. **(b)** Western blot showed Tet1 and Tet2 depletion in BMMSCs derived from *Tet* DKO mice. **(c)** Flow cytometric analyzed the expression of CD105, CD90, CD73, PDGFRα, Sca1, CD34 and CD45 in control and *Tet* DKO BMMSCs. **(d, e)** When cultured under adipogenic inductive conditions, the capacities to form adipocytes of control and *Tet* DKO BMMSCs were evaluated by Oil-red O staining **(d)**, and the expression of adipogenic markers *PPARγ* and *LPL* were analyzed by PCR **(e)**. **(f)** Western blot analyzed the efficacy of Tet1 and Tet2 siRNA knockdown in BMMSCs. **(g)** The proliferation rate in control and Tet1 and Tet2 siRNA treated-BMMSCs, as assessed by BrdU labeling assay. **(h, i)** The mineralized nodule formation and expression of osteogenic markers *Runx2*, *ALP* and *OCN* of control and Tet1 and Tet2 siRNA treated-BMMSCs, as assessed by alizarin red staining (**h**) and Western blotting **(i)**. **(j)** New bone formation of control and Tet1 and Tet2 siRNA treated BMMSCs (*B*) when subcutaneously implanted into immunocompromised mice using hydroxyapatite tricalcium phosphate (HA/TCP; *HA*) as a carrier for 8 weeks. $^{**}P < 0.01$. (mean ± SD). Scale bar, 50 μm. Results are from three independent experiments.

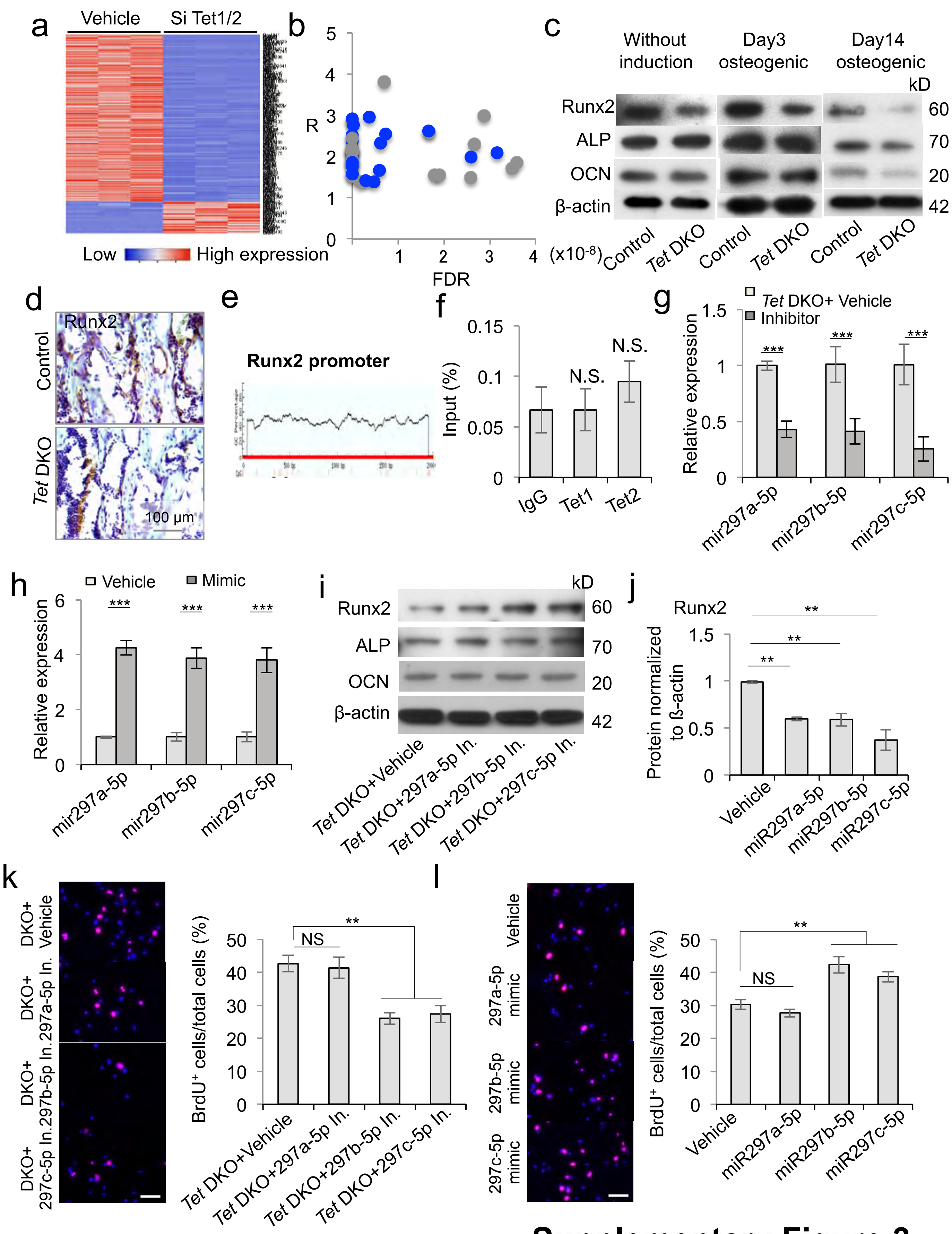

Supplementary Figure 3

Supplementary Figure 3. Related to Figure 4: **miR-297a-5p, miR-297b-5p and miR-297c-5p accumulate in *Tet* DKO BMMSCs to regulate *Runx2* signaling. (a)** The heatmap showed the relative gene expression levels in control and Tet1 and Tet2 siRNA treated BMMSCs. **(b)** The functional phenotype related to the genes altered more than 2-fold were analyzed by WebGestalt. Each dot in the plot shows an enrichment of phenotype category. The blue dot represents the 19-phenotype categories, which related to skeletal bone/cartilage development and morphology. **(c)** The expression of *Runx2*, *ALP* and *OCN* in control and *Tet* DKO BMMSCs under 0 day, 3 days and 14 days-osteogenic induction. **(d)** Runx2 positive cells were detected in control and *Tet* DKO mouse bone marrow by immunohistological staining. **(e)** No CpG island was founded in *Runx2* promoter. **(f)** Tet1 and Tet2 have no binding on the promoter of *Runx2* in BMMSCs, as assessed by ChIP-qPCR. IgG was used as a control. **(g)** The efficacy of miR-297a-5p, miR-297b-5p and miR-297c-5p inhibitor treatment in *Tet* DKO BMMSCs, as assessed by qPCR analysis. **(h)** The efficacy of miR-297a-5p, miR-297b-5p and miR-297c-5p mimic treatment in BMMSCs, as assessed by qPCR analysis. **(i)** Western blotting showed the expression of *Runx2*, *ALP* and *OCN* in *Tet* DKO and miR-297a-5p, miR-297b-5p and miR-297c-5p inhibitor treated *Tet* DKO BMMSCs under 3 days osteogenic induction. **(j)**The quantification of protein expression level according to Fig. 4i. **(k)** BrdU labeling assay showed the proliferation rate of *Tet* DKO and miR-297a-5p, miR-297b-5p and miR-297c-5p inhibitor treated *Tet* DKO BMMSCs. **(l)** BrdU labeling assay showed the proliferation rate of control and miR-297a-5p, miR-297b-5p and miR-297c-5p mimic treated BMMSCs. $^{**}P< 0.01$, $^{***}P< 0.001$. (mean ± SD). Scale bars, 100 μm (d),50 μm (k, l). Results are from three independent experiments.

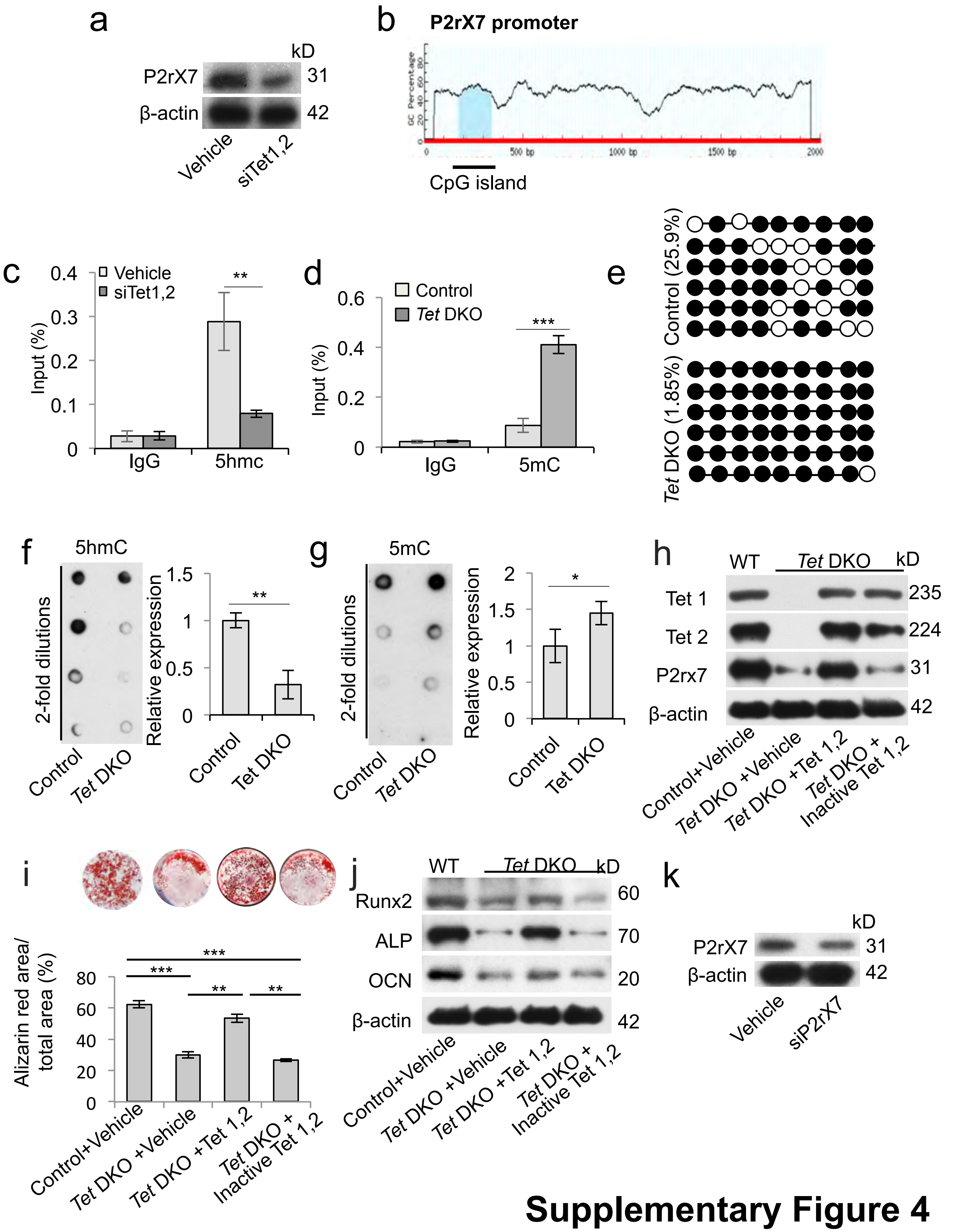

Supplementary Figure 4

Supplementary Figure 4. Related to Figure 5: **Tet1 and Tet2 control microRNA secretion through demethylation of *P2rX7* promoter in BMMSCs**. **(a)** The expression of *P2rX7* in control and Tet1 and Tet2 siRNA treated-BMMSCs, as assessed by Western blotting. **(b)** The CpG island of P2rX7. **(c)** Enrichment of 5hmC at the *P2rX7* promoter in control and Tet1 and Tet2 siRNA treated-BMMSCs, as assessed by hMeDIP-qPCR analysis. IgG was used as a control. **(d)** Enrichment of 5mC at the *P2rX7* promoter in control and Tet1 and Tet2 siRNA treated-BMMSCs, as assessed by MeDIP-qPCR analysis. IgG was used as a control. **(e)** Ox-BS sequencing analysis showed that *Tet* DKO BMMSCs displayed decreased hydroxymethylation and elevated methylation in the promoter of P2rX7 locus compared to control BMMSCs. Each dot represents a single CpG. Empty dots indicate unmethylated CpGs; Black dots indicate methylated CpGs. **(f)** Global 5hmC expression in control and *Tet* DKO BMMSCs, as assessed by dot blot assay. **(g)** Global 5mC expression in control and *Tet* DKO BMMSCs, as assessed by dot blot assay. **(h)** Western blotting showed wildtype Tet1 and Tet2 plasmid, but not catalytic domain inactive ones, overexpression rescued decreased expression of P2rX7 in *Tet* DKO BMMSCs. **(i, j)** Wildtype Tet1 and Tet2 plasmid, but not catalytic domain inactive ones, treated BMMSCs showed increased mineralized nodule formation as assessed by alizarin red staining **(i)**, and increased expression of osteogenic markers *Runx2*, *ALP* and *OCN* as assessed by Western blotting, **(j)**. **(k)** *P2rX7* siRNA knockdown efficacy in BMMSCs was shown by Western blotting. *$P<0.05$, **$P< 0.01$, ***$P< 0.001$. (mean ± SD). Results are from three independent experiments.

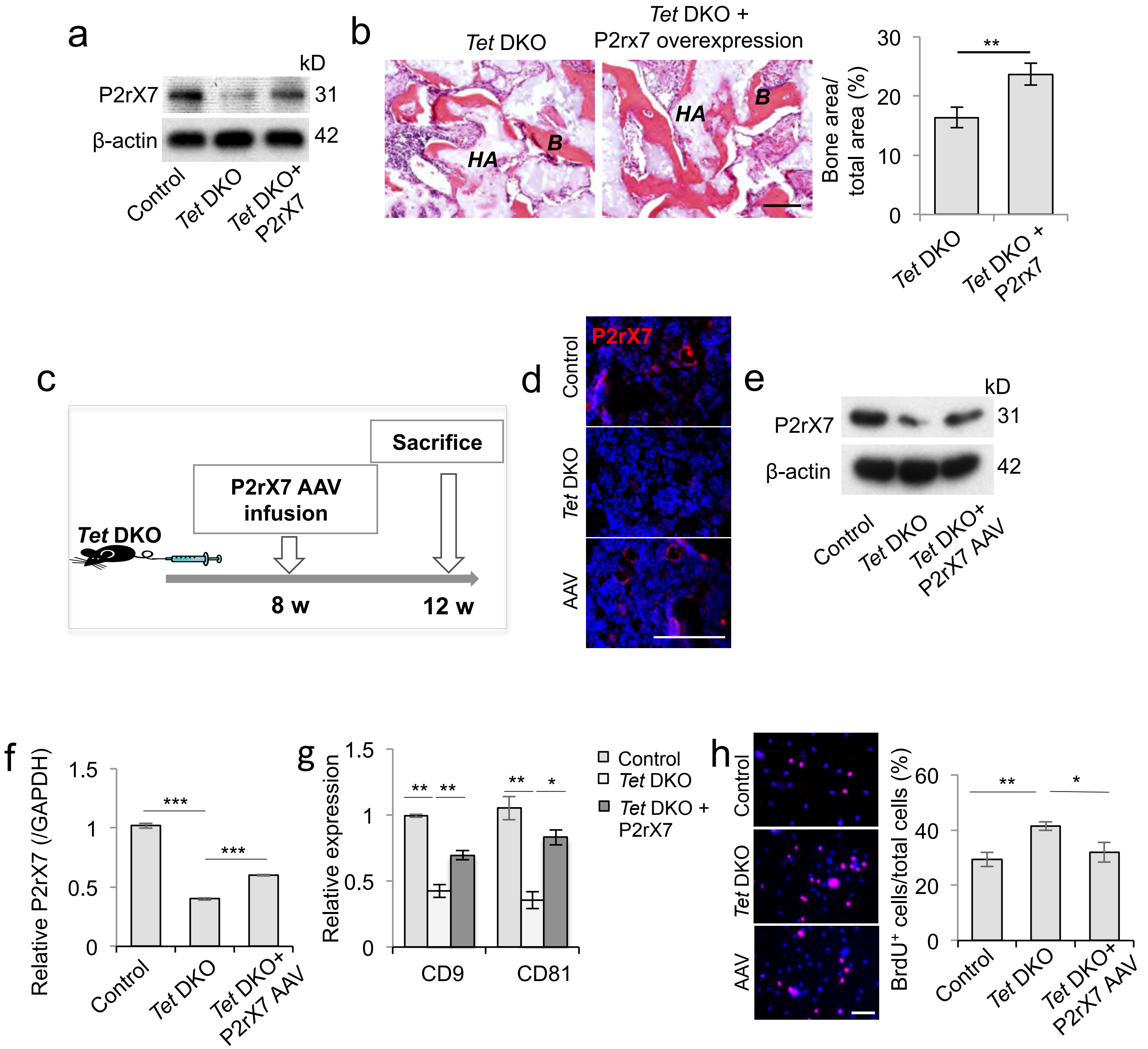

Supplementary Figure 5

Supplementary Figure 5. Related to Figure 6: ***P2rX7* activation rescues impairment of *Tet* DKO BMMSCs and osteopenia phenotype in *Tet* DKO mice**. **(a)** Western blot analysis showing the *P2rX7* CRISPR plasmid activation efficacy in *Tet* DKO BMMSCs. **(b)** New bone formation (*B*) of *Tet* DKO BMMSCs and *P2rX7* CRISPR activation plasmid treated *Tet* DKO BMMSCs when subcutaneously implanted into immunocompromised mice using hydroxyapatite tricalcium phosphate (HA/TCP; *HA*) as a carrier for 8 weeks. **(c)** Schema describing the use of Adeno-associated *P2rX7* overexpression virus (*P2rX7* AAV) to treat *Tet* DKO mice (n=5 per group). **(d)** The expression of *P2rX7* was observed in bone marrow cells of control, *Tet* DKO and *P2rX7* AAV-treated *Tet* DKO mice through immunostaining. **(e**, **f)** The expression of *P2rX7* in control, *Tet* DKO and *P2rX7* AAV treated-*Tet* DKO BMMSCs analyzed by Western blot (**e**) and qPCR (**f**). **(g)** The quantification of protein expression level according to Fig.6h. **(h)** BrdU labeling assay showed the proliferation rates of control, *Tet* DKO and *P2rX7* AAV treated-*Tet* DKO BMMSCs. Age of 8-10 weeks $Tet1^{-/-}Prx1^{cre}Tet2^{fl/fl}$ mice were used as *Tet* DKO mice in these experiments, and their female littermates which genetic status was $Prx1^{cre}$ were used as controls. *P<0.05, **P< 0.01, ***P< 0.001. (mean ± SD). Scale bars, 25 μm (d), 50 μm (b, h). Results are from three independent experiments.

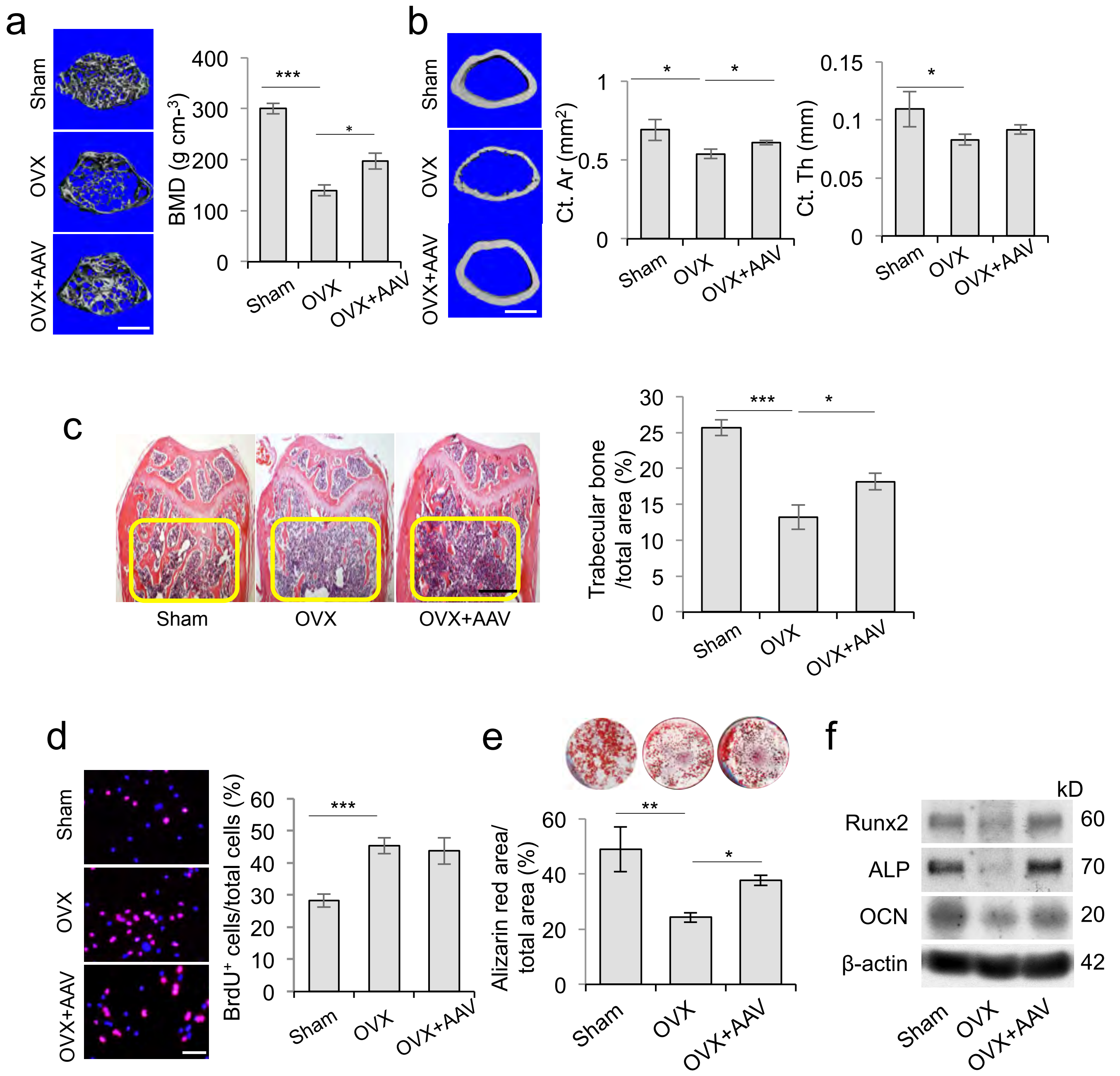

Supplementary Figure 6

Supplementary Figure 6. *P2rX7* activation rescues osteopenia phenotype in OVX mice. **(a)** Bone mineral density (BMD) of trabecular bone (TB) area in distal femurs of sham, OVX and *P2rX7* AAV treated OVX mice, as assessed by Micro-CT. **(b)** Micro-CT analysis showed that the cortical bone volume in the femur of sham, OVX and *P2rX7* AAV treated OVX mice, as determined by cortical bone area (Ct.Ar) and cortical thickness (Ct.Th) of the femur. (**c**) H&E staining showed trabecular bone volume (yellow-circled area) in the distal femurs of sham, OVX and *P2rX7* AAV treated OVX mice **(d)** BrdU labeling assay showed the proliferation rate of sham, OVX and *P2rX7* AAV treated OVX BMMSCs. **(e**, **f)** When cultured under osteogenic inductive conditions, the mineralized nodules formation and the expression of osteogenic markers *Runx2*, *ALP* and *OCN*, as assessed by alizarin red staining (**e**) and Western blotting (**f**). Age of 8-10 weeks C57BL/6J mice was used in these experiments. *P<0.05, **P< 0.01, ***P< 0.001. (mean ± SD). Scale bars, 400 μm (a, b), 1mm (c), 50 μm (d). Results are from three independent experiments.

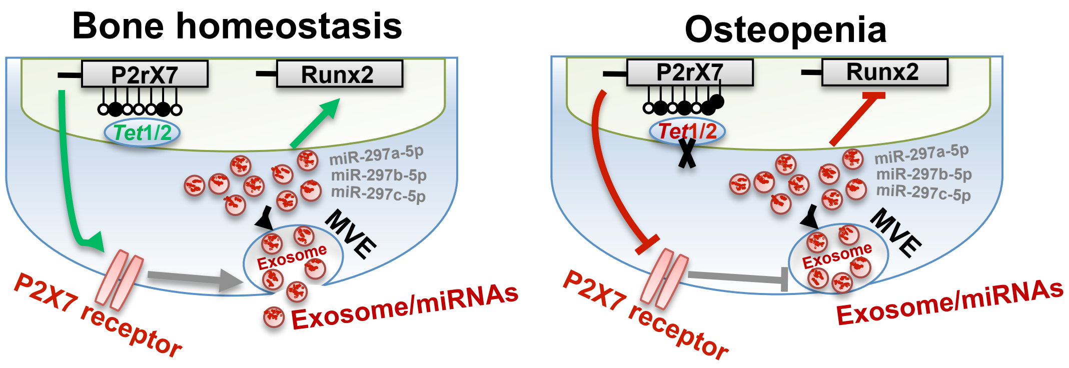

Supplementary Figure 7. Schematic diagram of Tet1 and Tet2 maintain BMMSC homeostasis *via* demethylation of *P2rX7*. Tet1/2 depletion-mediated hypermethylation of the *P2rX7* promoter to block miR-297a-5p, miR-297b-5p, and miR-297c-5p release, leading to downregulation of Runx2 signaling and osteopenia phenotype. MVE: Multivesiclar endosomes.

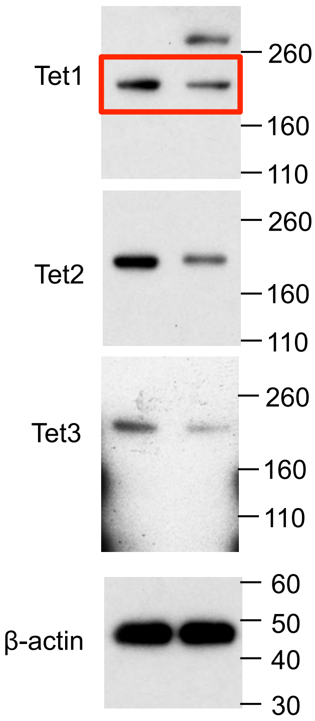

Fig. 1a

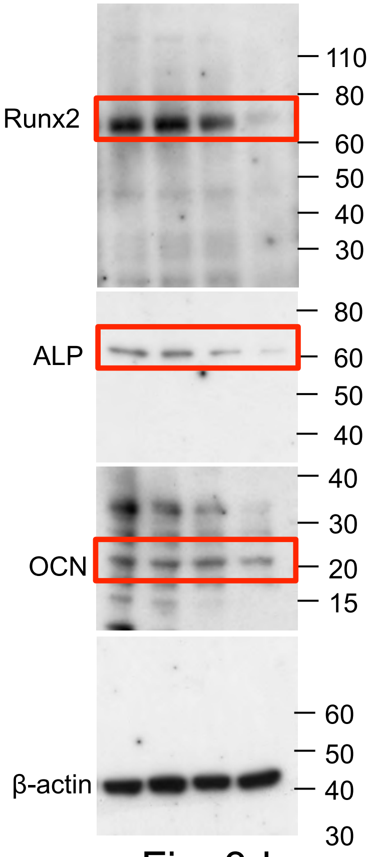

Fig. 3d

Supplementary Figure 8. Ucropped western blot images for Figure 1 and 3

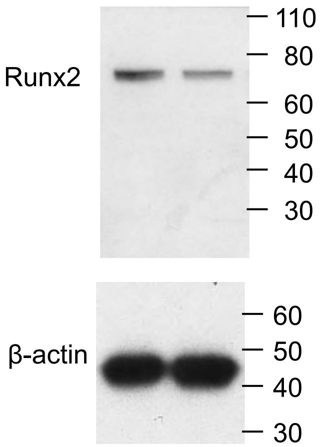

Fig. 4a

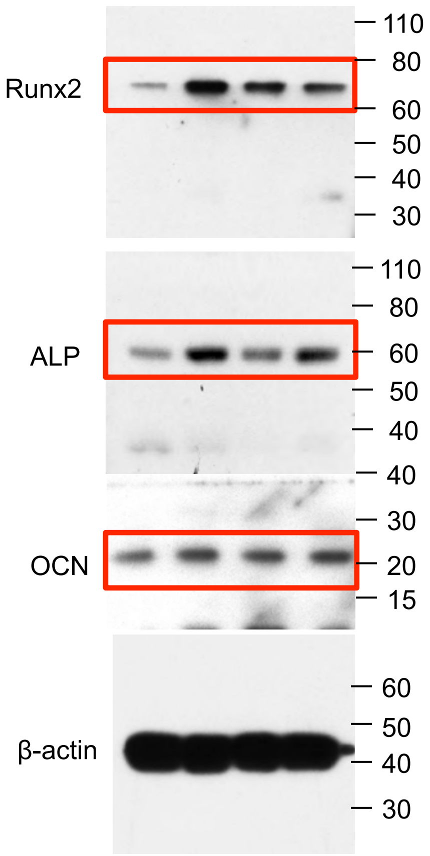

Fig. 4f

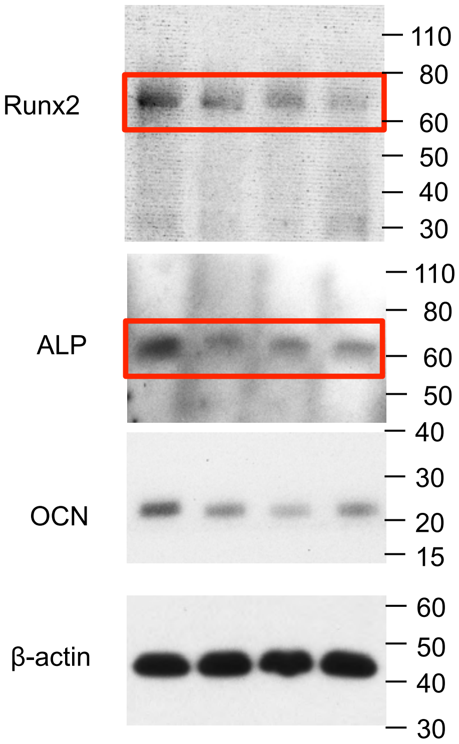

Fig. 4i

Supplementary Figure 9. Ucropped western blot images for Figure 4

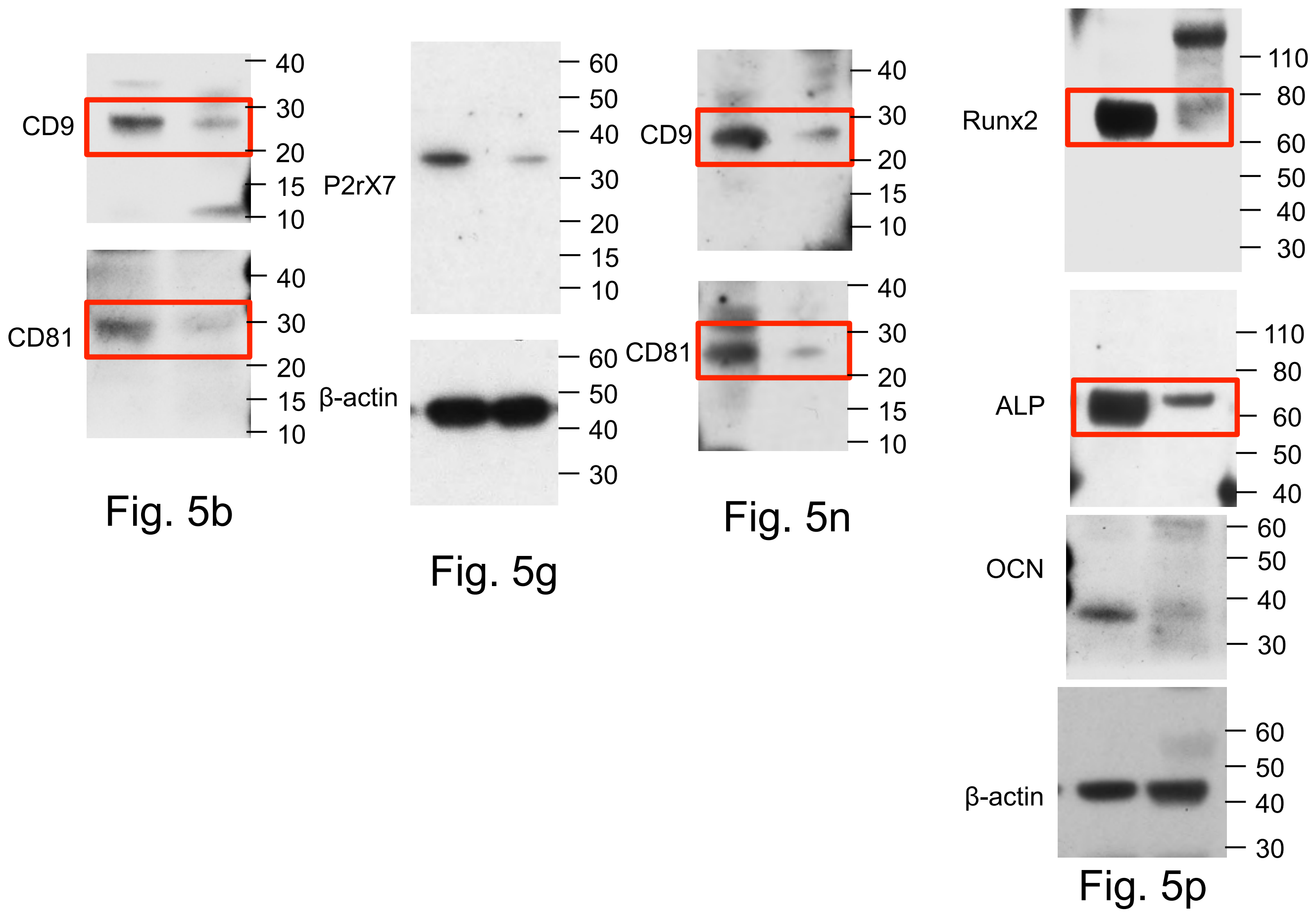

Supplementary Figure 10. Ucropped western blot images for Figure 5

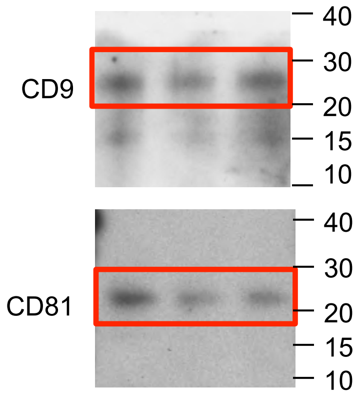

Fig.6c

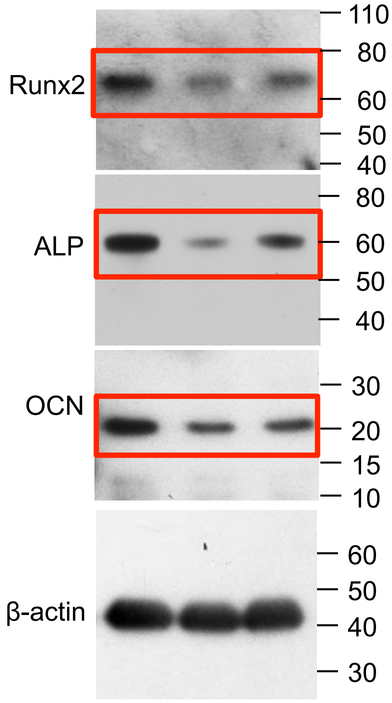

Fig.6e

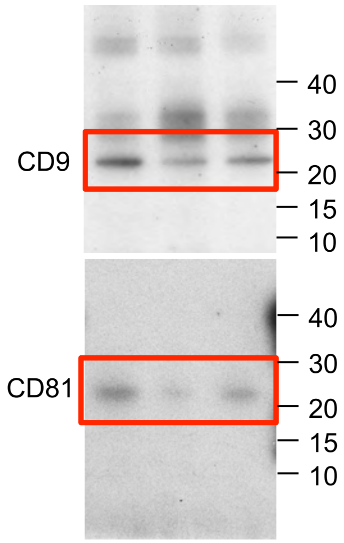

Fig.6h

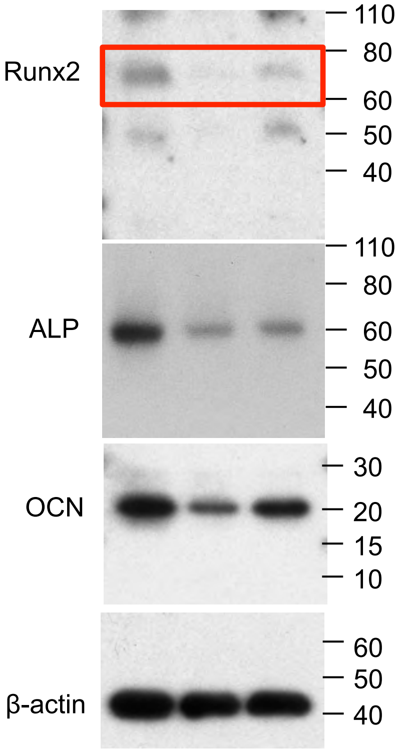

Fig. 6n

Supplementary Figure 11. Ucropped western blot images for Figure 6

Supplementary Table 1. miRNA hits on *Runx2*

<table>
<tr><th>Rfam ID</th><th>Start</th><th>End</th><th>Alignment</th></tr>
<tr><td>mmu-miR-293*</td><td>710</td><td>731</td><td>GUUUUACAGUGUGUCAAACUCA
|| |||| :|:| ||||||||
CATAATGGTATAGTGTTTGAGT</td></tr>
<tr><td>mmu-miR-297b-5p</td><td>44</td><td>65</td><td>UGUACAAGUACGUGUGUAUGUA
|::|||| || :|:|||||:||
ATGTGTTAATATATACATATAT</td></tr>
<tr><td>mmu-miR-710</td><td>472</td><td>493</td><td>UUGAGAGGGGUUCUGAAC
|::|:|||:: |||||||
AGTTTTCCTTCAGACTTG</td></tr>
<tr><td>mmu-miR-297c</td><td>44</td><td>65</td><td>UGUACAUGUACGUGUGUAUGUA
|::||| || :|:|||||:||
ATGTGTTAATATATACATATAT</td></tr>
<tr><td>mmu-miR-469</td><td>398</td><td>423</td><td>CCUGUGGUUCUAGUUACUUUCUCCGU
| :|||: ||| :: ||||||||
GTGCACTCAGAAAGGGACAAGAGGCA</td></tr>
<tr><td>mmu-miR-297a</td><td>44</td><td>65</td><td>UGUACGUGUACGUGUGUAUGUA
|::||: || :|:|||||:||
ATGTGTTAATATATACATATAT</td></tr>
<tr><td>mmu-miR-101a*</td><td>151</td><td>172</td><td>CGUAGUCGUGACACUAUUGACU
| :| :| :| || |||||||:
GGGTGGGGGCAGTCATAACTGG</td></tr>
<tr><td>mmu-miR-330*</td><td>311</td><td>333</td><td>GGGACACGAAAC
|:||||||||||
CTCTGTGCTTTG</td></tr>
<tr><td>mmu-miR-466a-5p</td><td>45</td><td>66</td><td>AUACAUGUACAUGUGUGUGUAU
|:||| || ||:|||:|:|||
TGTGTTAATATATACATATATA</td></tr>
<tr><td>mmu-miR-872</td><td>241</td><td>261</td><td>GGACUUGAUUGUUCAUUGGA
:||| :| |:||||:||||
TCTGTTTTCATAAGTGACCT</td></tr>
<tr><td>mmu-miR-453</td><td>337</td><td>361</td><td>GUUCGAG-UGAUACUCCGUUGG
||: ||| :||:|| ||||:||
CAGCCTCTGCTGTGTGGCAGCC</td></tr>
<tr><td>mmu-miR-383</td><td>222</td><td>243</td><td>CGGUGUCAGUGGAAGACUAGA
|:|| || :::: ||||:|||
GTCAAAGGTGTTGTCTGGTCT</td></tr>
<tr><td>mmu-miR-666-3p</td><td>354</td><td>376</td><td>UCGUCCGCUA-GUGCGACGUC
:|||| ||:| ||| ||||||
GGCAGCCGGTACACTCTGCAG</td></tr>
<tr><td>mmu-miR-466d-3p</td><td>69</td><td>89</td><td>CACACGCACAUACAUAU
||| : | ||||||||
GTGCCTATATATGTATA</td></tr>
<tr><td>mmu-miR-369-3p</td><td>726</td><td>746</td><td>UAGUUGGUACAUAAUA
:|:::|::|||:||||
GTTGGCTGTGTGTTAT</td></tr>
<tr><td>mmu-miR-30b</td><td>167</td><td>189</td><td>UCGACUCACA-UCCUACAAAUG
| |||:|| | | |||||||
AACTGGGTTTCATATTGTTTAC</td></tr>
<tr><td>mmu-miR-411</td><td>170</td><td>190</td><td>GCAUGCGAUAUGCCAGAUGAU
:| :: : |||: ||:|||||
TGGGTTTCATATTGTTTACTA</td></tr>
<tr><td>mmu-miR-379</td><td>168</td><td>190</td><td>GAUGCAAGGUAU--CAGAUGGU
||: |||:|||| ||:|||:|
CTGGGTTTCATATTGTTTACTA</td></tr>
<tr><td>mmu-miR-770-5p</td><td>381</td><td>402</td><td>UGUGCACCACG
| |:|||||||
AGATGTGGTGC</td></tr>
</table>

Supplementary Table 2. Related to Experimental Procedures. Primer sequences used for PCR to quantify expression of various genes (qPCR), ChIP-PCR and P2rX7 bisulfite sequencing. Forward (F) and reverse (R) primers are used for analysis

**qPCR**	**Primer**
mTet1 F	TGG GAG AGC TCC CTT TGA TGG TTT
mTet1 R	TTG GGT CAA TTG TGC TGC GAC ATC
mTet2 F	TGT TGT TGT CAG GGT GAG AAT C
mTet2 R	TCT TGC TTC TGG CAA ACT TAC A
mTet3 F	AAC CAG AAC GCC AAG GTC AGT AGT
mTet3 R	TTG ATC TTC TCT GGC GTG CTC AGT
hTet1 F	CAG AAC CTA AAC CAC CCG TG
hTet1 R	TGC TTC GTA GCG CCA TTG TAA
hTet2 F	GGC TAC AAA GCT CCA GAA TGG
hTet2 R	AAG AGT GCC ACT TGG TGT CTC
hTet3 F	TCC AGC AAC TCC TAG AAC TGA G
hTet3 R	AGG CCG CTT GAA TAC TGA CTG
mP2rX7 F	CAG CGG AAA GAG CCT GTT ATC
mP2rX7 R	TGG CCT TCT GAC TTG ACA TAG TT
mRunx2 F	AGA GTC AGA TTA CAG ATC CCA GG
mRunx2 R	TGG CTC TTC TTA CTG AGA GAG G
mALP F	GGC TGG AGA TGG ACA AAT TCC
mALP R	CCG AGT GGT AGT CAC AAT GCC
mOCN F	CTG ACC TCA CAG ATC CCA AGC
mOCN R	TGG TCT GAT AGC TCG TCA CAA G
**ChIP-PCR**	
mP2rX7 promoter F	AAA GGT CAG GGC AGA GTT AAG
mP2rX7 promoter R	CAT GTG GTT GCT CGG ATT TG
mRunx2 promoter F	ACC TAG GCA GAA GGT CAT ACA
mRunx2 promoter R	AGC ATC CAT GTT CCA ACT CAA
**miRNA**	
mmu-miR-293*	CGC AGG TTT TAC AGT GTG TC
mmu-miR-297b-5p	CGC AGA TGT ATG TGT GC
mmu-miR-710	CCA AGT CTT GGG GAG AGT
mmu-miR-297c	CGC AGA TGT ATG TGT GC

mmu-miR-469	CCT GTG GTT CTA GTT ACT TTC TC
mmu-miR-297a	GAT GTA TGT GTG CAT GTG C
mmu-miR-101a*	GCA GTC AGT TAT CAC AGT GCT
mmu-miR-330*	GAG ACG TCC GGG ACA
mmu-miR-872	CGC AGA AGG TTA CTT GTT AG
mmu-miR-453	GGT TCG AGT GAT ACT CCG T
mmu-miR-383	CAG AGA TCA GAA GGT GAC TGT
mmu-miR-666-3p	GCA GCG TGA TCG CCT
mmu-miR-466d-3p	CAG TAT ACA TAC ACG CAC ACA
mmu-miR-369-3p	GCG CAG TTT CTA GTT GGT
mmu-miR-30b	GCA GTG TAA ACA TCC TAC ACT CA
mmu-miR-411	GGC ATG CGA TAT GCC A
mmu-miR-379	GCA GGG ATG CAA GGT ATC
mmu-miR-770-5p	CGC ACC GGG TCT GTG
**Bisulfite sequencing**	
P2rX7 promoter F	TTT ATT GGT TTT ATT TAT TGA AGG T
P2rX7 promoter R	TCT CCC AAC CCT CTC TAT TTA C
**Mouse genotyping**	
oIMR1084 Prrx1	GCG GTC TGG CAG TAA AAA CTA TC
oIMR1085 Prrx1	GTG AAA CAG CAT TGC TGT CAC TT
13037 Tet1	TCA GGG AGC TCA TGG AGA CTA
13038 Tet1	TTA AAG CAT GGG TGG GAG TC
13039 Tet1	AAC TGA TTC CCT TCG TGC AG
13495 Tet2	AAG AAT TGC TAC AGG CCT GC
13496 Tet2	TTC TTT AGC CCT TGC TGA GC
**RT-PCR primer**	
PPARY F	GCT GTT ATG GGT GAA ACT CTG
PPARY R	ATA AGG TGG AGA TGC AGG TTC
LPL F	GGG CTC TGC CTG AGT TGT AG
LPL R	AGA AAT TTC GAA GGC CTG GT

Supplementary table 3. Categories of phenotype determined by WebGestalt. The 40 most significant categories of phenotype determined by WebGestalt analysis using the altered genes between Vehicle and siTet1,2 BMMSCs group.

ID	Name
MP:0000163	abnormal cartilage morphologyC=286; R=2.52; PValue=0e+00; FDR=0e+00
MP:0000556	abnormal hindlimb morphologyC=331; R=2.37; PValue=0e+00; FDR=0e+00
MP:0002113	abnormal skeleton developmentC=394; R=2.19; PValue=1.11e-16; FDR=2.22e-13
MP:0001533	abnormal skeleton physiologyC=541; R=1.98; PValue=2.22e-16; FDR=3.33e-13
MP:0005274	abnormal viscerocranium morphologyC=409; R=2.12; PValue=8.88e-16; FDR=1.07e-12
MP:0000454	abnormal jaw morphologyC=354; R=2.19; PValue=3.66e-15; FDR=3.66e-12
MP:0002115	abnormal limb bone morphologyC=393; R=2.11; PValue=6e-15; FDR=5.14e-12
MP:0003856	abnormal hindlimb stylopod morphologyC=152; R=2.91; PValue=8.22e-15; FDR=6.16e-12
MP:0002109	abnormal limb morphologyC=715; R=1.77; PValue=2e-14; FDR=1.33e-11
MP:0002116	abnormal craniofacial bone morphologyC=584; R=1.86; PValue=2.8e-14; FDR=1.68e-11
MP:0001614	abnormal blood vessel morphologyC=955; R=1.64; PValue=3.42e-14; FDR=1.74e-11
MP:0011504	abnormal limb long bone morphologyC=376; R=2.1; PValue=3.47e-14; FDR=1.74e-11
MP:0008271	abnormal bone ossificationC=340; R=2.17; PValue=3.93e-14; FDR=1.81e-11
MP:0009250	abnormal appendicular skeleton morphologyC=616; R=1.83; PValue=4.51e-14; FDR=1.93e-11
MP:0000438	abnormal cranium morphologyC=572; R=1.86; PValue=5.91e-14; FDR=2.36e-11
MP:0000559	abnormal femur morphologyC=150; R=2.84; PValue=7.46e-14; FDR=2.79e-11
MP:0005369	muscle phenotypeC=1094; R=1.58; PValue=7.89e-14; FDR=2.79e-11
MP:0000130	abnormal trabecular bone morphologyC=229; R=2.44; PValue=9.75e-14; FDR=3.25e-11
MP:0003723	abnormal long bone morphologyC=482; R=1.93; PValue=1.43e-13; FDR=4.46e-11
MP:0001544	abnormal cardiovascular system physiologyC=1299; R=1.52; PValue=1.49e-13; FDR=4.46e-11
MP:0000550	abnormal forelimb morphologyC=149; R=2.76; PValue=8.71e-13; FDR=2.49e-10
MP:0005390	skeleton phenotypeC=1854; R=1.38; PValue=6.91e-12; FDR=1.88e-09
MP:0002127	abnormal cardiovascular system morphologyC=1548; R=1.42; PValue=1.13e-11; FDR=2.94e-09
MP:0004986	abnormal osteoblast morphologyC=110; R=2.96; PValue=1.48e-11; FDR=3.71e-09
MP:0005508	abnormal skeleton morphologyC=1752;R=1.39; PValue=1.98e-11; FDR=4.75e-09
MP:0002108	abnormal muscle morphologyC=726; R=1.66; PValue=2.58e-11; FDR=5.94e-09
MP:0003857	abnormal hindlimb zeugopod morphologyC=214; R=2.32; PValue=2.78e-11; FDR=6.17e-09
MP:0000088	short mandibleC=57; R=3.81; PValue=3.24e-11; FDR=6.93e-09
MP:0006395	abnormal epiphyseal plate morphologyC=162; R=2.54; PValue=3.55e-11; FDR=7.34e-09
MP:0000458	abnormal mandible morphologyC=142; R=2.62; PValue=8.35e-11; FDR=1.67e-08
MP:0000164	abnormal cartilage developmentC=147; R=2.59; PValue=9.33e-11; FDR=1.79e-08
MP:0000428	abnormal craniofacial morphologyC=938; R=1.55; PValue=9.53e-11; FDR=1.79e-08
MP:0005382	craniofacial phenotypeC=939; R=1.54; PValue=1.05e-10; FDR=1.9e-08
MP:0005388	respiratory system phenotypeC=1110; R=1.49; PValue=1.49e-10; FDR=2.59e-08
MP:0000249	abnormal blood vessel physiologyC=327; R=1.99; PValue=1.51e-10; FDR=2.59e-08
MP:0000074	abnormal neurocranium morphologyC=207; R=2.29; PValue=1.6e-10; FDR=2.66e-08
MP:0004592	small mandibleC=96; R=2.99; PValue=1.77e-10; FDR=2.87e-08
MP:0004686	decreased length of long bonesC=275; R=2.09; PValue=2e-10; FDR=3.16e-08
MP:0003743	abnormal facial morphologyC=570; R=1.71; PValue=2.27e-10; FDR=3.48e-08
MP:0000452	abnormal mouth morphologyC=419; R=1.85; PValue=2.4e-10; FDR=3.59e-08
